# Cerebral Venous Thrombosis as an Initial Presentation of Ulcerative Colitis

**DOI:** 10.1155/2022/9438757

**Published:** 2022-03-27

**Authors:** Hiroshi Yamazaki, Akinori Sasaki, Eriko Yamaguchi, Kana Sawada, Risa Okamoto, Kuniyasu Saigusa, Yasuaki Motomura

**Affiliations:** ^1^Department of Gastroenterology, Tokyo Bay Urayasu Ichikawa Medical Center, 3-4-32 Toudaijima, Urayasu, 279-0001 Chiba, Japan; ^2^Department of Neurosurgery, Tokyo Bay Urayasu Ichikawa Medical Center, 3-4-32 Toudaijima, Urayasu, 279-0001 Chiba, Japan

## Abstract

Cerebral venous thrombosis (CVT) is a rare complication of ulcerative colitis (UC) that is potentially fatal once it occurs. This report describes a case of CVT that led to a diagnosis of UC. A 48-year-old woman was diagnosed with CVT due to paresthesia and weakness and was hospitalized for treatment. She developed bloody diarrhea on admission and was further diagnosed with UC based on endoscopic and pathologic findings. Treatment of UC with steroids and sulfasalazine was administered immediately. Her condition improved significantly within several days following treatment. After discharge, the patient experienced no recurrence of either CVT or UC flare-up over the last five years. This report describes CVT as an initial presentation of UC. This is also the first report of a long-term follow-up following successful treatment of CVT with concomitant UC.

## 1. Introduction

Ulcerative colitis (UC) is a chronic disease characterized by diffuse inflammation of the rectal and colonic mucosa. In recent decades, the incidence of UC has significantly increased in several regions of the world, including Japan [1].

The clinical symptoms of UC include bloody diarrhea, abdominal pain, and tenesmus [2]. Moreover, UC may also present as a systemic disease accompanied by various extraintestinal manifestations. Thromboembolic events, such as deep venous thrombosis and pulmonary embolism, are common manifestations of UC [3]. On the other hand, cerebral venous thrombosis (CVT) is a rare but potentially fatal extraintestinal manifestation of UC [4].

A few cases of CVT due to UC have been reported in the literature, and most of these described the onset of CVT during an acute exacerbation of UC [5]. Simultaneous occurrence of CVT and UC is extremely rare in patients newly diagnosed with UC. This report describes our experience with a patient who presented with CVT, leading to a diagnosis of UC.

## 2. Case Presentation

A 48-year-old woman was admitted to our hospital with paresthesia and left upper extremity weakness for two days. The patient had a history of occasional bloody stools in the past few months before admission, which were assumed due to hemorrhoids. In addition, she had a recent history of nonbloody, loose stools in the past five days prior to admission. The patient was completely blind due to bilateral enucleation performed for retinoblastoma at one year of age. On physical examination, she was febrile (37.7°C) with pulse rate of 94/min and blood pressure of 132/86 mmHg. Her abdomen was flat with hyperactive bowel sounds and no abdominal tenderness. Neurological examination revealed muscle weakness of the left extremity with a manual muscle testing score of 3/5 and 4/5 in the left upper extremity and left lower extremity, respectively.

Laboratory test results showed low hemoglobin levels of 10 g/dl and a white blood cell count of 5800/mm^3^. Erythrocyte sedimentation rate was 22 mm/h during the first hour. C-reactive protein level was elevated (3.26 mg/dl). Prothrombin time/international normalized ratio was 1.11 and activated partial thromboplastin time was 32.5 s. Liver and renal function tests and serum electrolyte levels were within the normal range.

**Figure 1:**
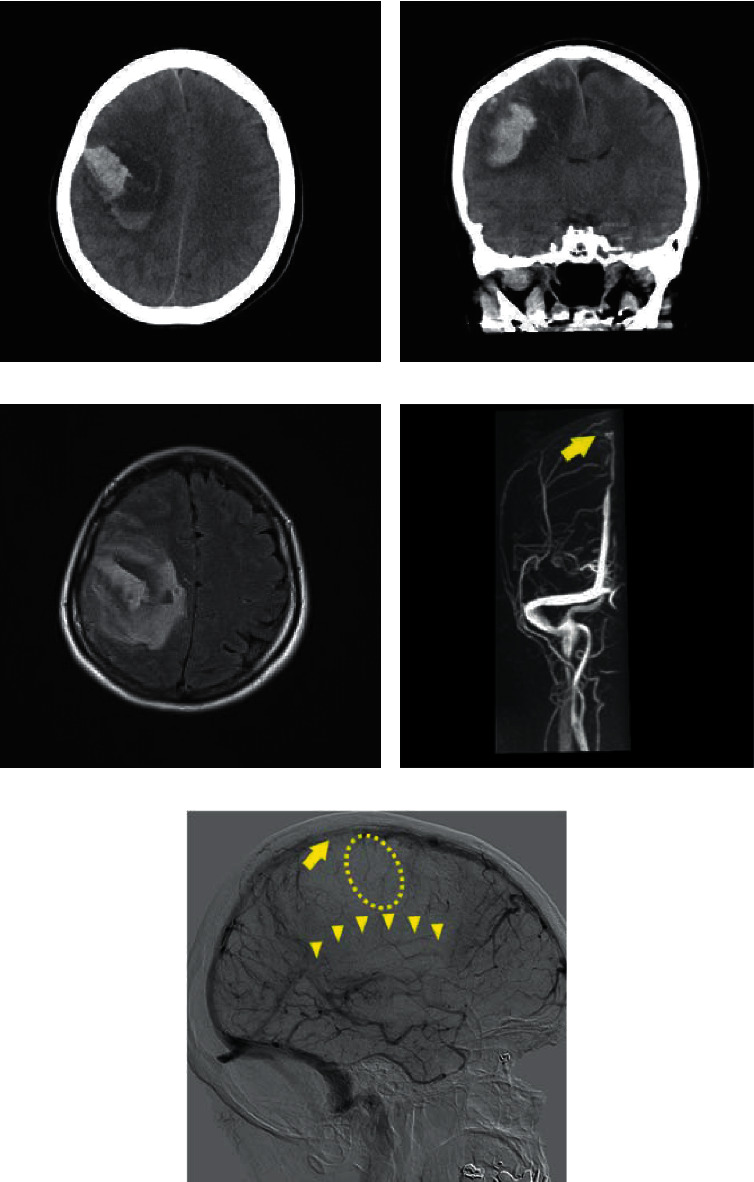
(a) Sagittal and (b) coronal images of nonenhanced brain computed tomography (CT) showing a high-density area in the right frontoparietal lobe, consistent with right lobar intracranial hemorrhage (ICH). (c) A head magnetic resonance image (MRI) with fluid attenuation inversion recovery (FLAIR) showing infarction surrounding subcortical hemorrhage of the frontoparietal lobe. (d) Magnetic resonance venography (MRV) revealing an absence of normal flow patterns of the superior sagittal sinus (arrow). (e) Cerebral angiogram showing total occlusion of the right rolandic vein (dotted circle) and inferior sagittal sinus (arrow heads) and partial occlusion of the superior sagittal sinus (arrow) caused by thrombosis (Figure 1(c)).

A computed tomography (CT) scan of the head revealed a high-density area in the right frontoparietal lobe (Figure 1(a)). Brain magnetic resonance imaging (MRI) showed an infarction complicated by hemorrhage in the same lobe (Figure 1(c)). In addition, cerebral angiography revealed total occlusion of the right rolandic vein and inferior sagittal sinus and partial occlusion of the superior sagittal sinus (Figure 1(e)).

The patient was diagnosed with cerebral venous sinus thrombosis. Surgical hematoma evacuation was performed immediately upon hospitalization, and unfractionated heparin was administered (20,000 units/day). Prothrombotic workup such as protein C and S and antithrombin III were within the normal range. Antinuclear antibodies and anticardiolipin antibodies were negative.

**Figure 2:**
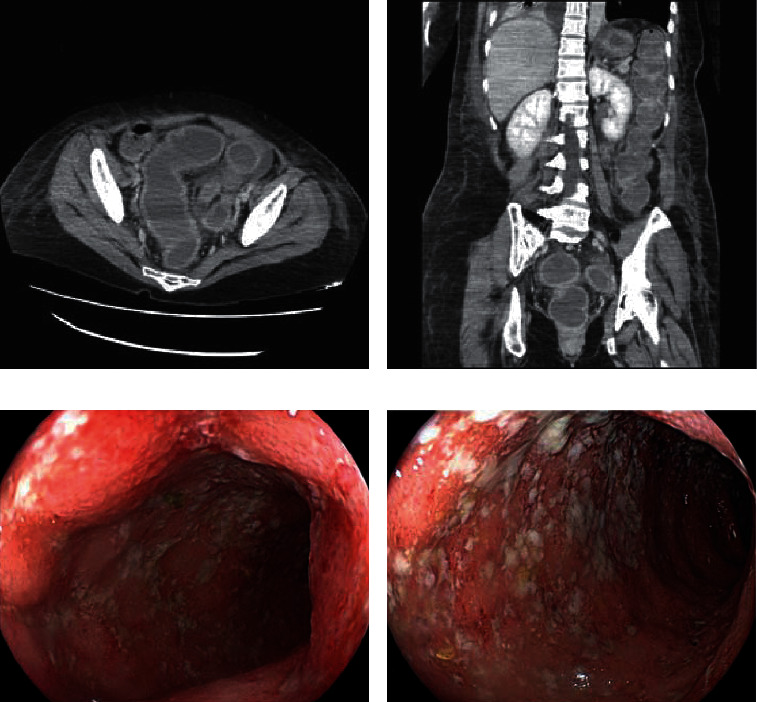
(a) Axial and (b) coronal images of contrast enhanced computed tomography (CT) showing colonic mural thickening and enhancement of the left-sided colon, consistent with colitis. Hyperattenuating material within the bowel lumen can be seen, suggestive of blood clots. Endoscopic findings of the (c) rectum and (d) sigmoid colon showing edematous mucosa with marked erythema, absent vascular pattern, friability, and erosions, which are consistent with ulcerative colitis (UC).

She was in a stable condition following surgery. However, she experienced fever and bloody diarrhea on day seven after admission. Abdominal CT scan demonstrated thickening of the bowel wall from the rectum to the descending colon (Figure 2(a)). Rapid sigmoidoscopy revealed edematous mucosa, erythema, and loss of vascular markings, which extended continuously from the rectum (Figure 2(c)). Neither longitudinal nor discrete ulcers were seen, which were suggestive of ischemic colitis. These endoscopic findings were consistent with ulcerative colitis, Mayo endoscopic subscore (MES) of 2, and confirmed by colonic biopsies that showed crypt abscesses and goblet cell loss. Stool culture was negative. The *Clostridium difficile* toxin A/B test and indirect immunofluorescence assay antibody titer for *Entamoeba histolytica* were negative. No antibiotics responsible for drug-induced colitis were administered.

She was administered steroids (prednisolone, 60 mg/day), sulfasalazine (4 g/day), and topical mesalamine (1 g/day). Within 5 days following treatment initiation, her digestive symptoms, diarrhea, and bloody stools resolved, and her temperature returned to normal. Prednisolone was tapered without any flare-ups, and her anticoagulant medication was switched from unfractionated heparin to warfarin.

**Figure 3:**
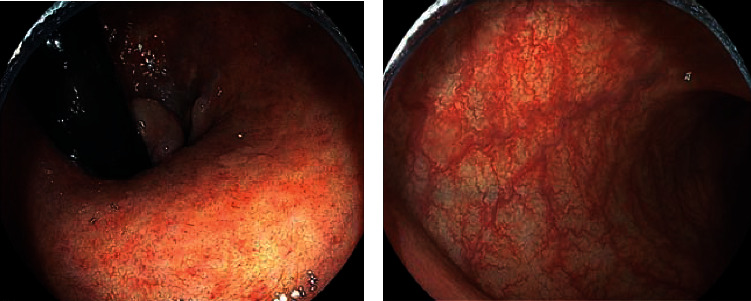
25 months after treatment, endoscopic findings of the (a) rectum and (b) sigmoid colon showing normal mucosa with visible vascular pattern, consistent with endoscopic remission, Mayo endoscopic subscore (MES) 0.

Her digestive and neurological symptoms showed significant improvement, and she was transferred to a rehabilitation hospital on day 70. By the time she was discharged from the rehabilitation hospital, clinical remission had been achieved, and her first total colonoscopy (TCS) was performed 9 months after the initial diagnosis. TCS revealed mild erythema and loss of vascular markings in mucosa of the descending and sigmoid colon and some erosions and mucosal friability in the rectum, which were consistent with MES of 1. No signs of inflammation were seen between the cecum and transverse colon, leading to a diagnosis of left-sided UC. Mesalamine suppository was added following TCS, and MES of 0 was confirmed by sigmoidoscopy 16 months later (Figure 3). Her UC has been well controlled for the last five years with mesalamine 4800 mg/d, mesalamine suppository 1 g/d, and probiotics, and her latest MES remained 0.

Warfarin was stopped after 12 months, and she experienced no recurrence of symptoms over the last five years after discontinuation of anticoagulation treatment.

## 3. Discussion

In the present case, the patient received treatment of both CVT and UC shortly after diagnosis, and her symptoms improved within a few days. In addition, she had an uneventful course without recurrence of CVT or UC flare-up for more than five years. To our best knowledge, this is the first report of a long-term follow-up after successful treatment of CVT with concomitant UC.

Generally, UC is a chronic idiopathic inflammatory bowel disorder (IBD) of the mucosal layer of the colon. It usually occurs in patients aged 20–30 years, with a second peak between the ages of 70 and 80 years. Patients with UC usually experience diarrhea, hematochezia, abdominal pain, tenesmus, and incontinence [2].

Furthermore, UC can be also regarded as a systemic disease with numerous extraintestinal complications. Patients with UC are at an increased risk of venous and arterial thromboembolism. Specifically, the risk of venous thromboembolism increases during UC flare-ups [6, 7]. Of these manifestations, thromboembolic events, such as deep venous thrombosis and pulmonary embolism, have been well recognized [3]. Although CVT is a rarely reported thromboembolic complication in patients with UC, it can be fatal.

It has been revealed that 0.5–6.4% of patients with UC experience CVT complications at some points in the course of their disease [8, 9]. Usually, CVT occurs in young patients with UC, with a mean age of <29 years [10]. This finding shows that patients with CVT and UC as comorbidity are significantly younger than those with CVT without UC. Cases of patients >40 years, such as the present case, are rarely reported in the literature. A previous report has shown that CVT was more common in patients with UC than in those with Crohn's disease, and female predominance has also been reported [10]. The prognosis of UC complicated with CVT was significantly worse than that of UC without CVT. One study demonstrated that the mortality rate in patients with UC with CVT could reach up to 25% [11]. The mechanism of thrombosis in UC is complex and incompletely understood [12]. However, a recent study has suggested an interaction between the coagulation cascade in the body and cytokine mediators of chronic inflammation with involvement of an inflammatory process in activating the coagulation cascade [13]. Coagulation activity in UC has been found to be related to disease activity and colonic extension of the disease [14]. Additionally, anemia, thrombocytosis, low albumin, and elevated D-dimer levels have been suggested as significant risk factors for CVT [10, 15]. Among these, severe iron deficiency anemia was identified as a significant risk factor for CVT in a previous study [16].

Although most case reports described CVT associated with flare-ups of established UC, this case was unique in which CVT preceded the diagnosis of UC. We suggest that in addition to chronic inflammation due to undiagnosed, hence, untreated UC, the patient experienced a flare-up in the past five days prior to admission when acute diarrhea had begun. Dehydration from diarrhea and iron deficiency were likely to have been the precipitating factors of the coagulation cascade, leading to CVT. Initially, the patient's relatively mild gastrointestinal symptom made the diagnosis of UC difficult as it seemed irrelevant to her neurological symptoms. Although physical and psychological stress due to CVT and surgery may have worsened the patient's flare-up, we hypothesize that administration of heparin ultimately provoked spontaneous bleeding from the inflamed membrane, leading to a definitive diagnosis.

The clinical manifestations of CVT comprise headache, uni or bilateral paresis, generalized or focal seizure, and encephalopathy. Headache is the most common symptom of venous sinus thrombosis, described in 89% of patients [17]. Seizure is also common in patients with CVT and reported in approximately 40% of these patients. Additionally, focal neurologic symptoms may reveal presence of venous infarction and hemorrhagic complication, which has been reported in more than one-third of patients [17].

Head CT and MRI are usually used for confirming CVT diagnosis. Moreover, CT without contrast is the initial imaging modality in patients with headaches and focal neurological symptoms [18]. Up to 30% of patients with venous sinus thrombosis would have an abnormal head CT scan without contrast. Generally, it may show hyperdensity of the cortical vein or dural venous sinus, which indicates infarct. In addition, approximately 30% of patients with CVT present with intracranial hemorrhage. Other imaging modalities such as CT venography and cerebral angiogram can assist in making a reliable diagnosis of CVT [18]. In this case, venous infarction in the superior and inferior sagittal sinuses was detected on a cerebral angiogram. However, the availability of these investigations may be influenced by institutional and regional factors and availability of specialists to perform these tests.

Currently, no guidelines are available for treatment of CVTs with concomitant UC [19]. However, several studies have shown that patients with IBD with CVT who received anticoagulation treatment showed better outcomes than those who did not [10, 20]. In addition, a previous study showed that timely diagnosis and treatment could result in a better prognosis in patients with CVT [21]. Despite concerns about hemorrhagic complications such as intestinal bleeding and intracranial hemorrhage, several studies have suggested improved survival in patients treated with anticoagulation, even in presence of intestinal bleeding and intracranial hemorrhage [10, 22]. Undifferentiated and low-molecular weight heparin is the most common drug used for prophylaxis and treatment of the acute phase of venous thromboembolism [23].

The efficacy and safety of long-term anticoagulant therapy in CVT with concomitant UC also remain unclear. The European Stroke Organization (ESO) recommends continuing anticoagulation for three months if CVT is due to a transient risk factor (e.g., pregnancy, dehydration, mechanical precipitants, and drugs) and for 6–12 months in patients with idiopathic CVT. On the other hand, these ESO guidelines recommend that anticoagulation therapy may be continued indefinitely in patients with recurrent CVT or CVT with severe thrombophilia. This severe thrombophilia is usually associated with homozygous prothrombin G20210 A mutations; homozygous factor V Leiden mutation; deficiencies in protein C, protein S, or antithrombin; combined thrombophilia defects; or antiphospholipid syndrome [24].

In presence of hypercoagulable conditions, CVT in patients with active UC should always be considered an indication for lifelong anticoagulation using warfarin [23]. However, it is unknown whether patients with CVT need to continue anticoagulant therapy after UC remission. In a previous study, CVT seldom occurred in patients with UC with stable remission [11]. In this case, warfarin was administered for 12 months and discontinued when the patient had a stable clinical course following clinical remission of UC. The patient has not experienced any recurrence of CVT without anticoagulant drugs use for more than five years.

In conclusion, in this report, we describe the case of new-onset UC after CVT. It is necessary to consider UC in patients with CVT, especially those with gastrointestinal symptoms. Early diagnosis and management of both UC and CVT may improve prognosis. Moreover, appropriate maintenance therapy for UC may prevent CVT recurrence. Further studies are warranted to further evaluate the optimal management and treatment of CVT with concomitant UC.

## Data Availability

The data used to support the findings of this study are available from the corresponding author upon request.
